# Validation of models for analysis of ranks in horse breeding evaluation

**DOI:** 10.1186/1297-9686-42-3

**Published:** 2010-01-28

**Authors:** Anne Ricard, Andrés Legarra

**Affiliations:** 1 INRA, UMR 1313, 78352 Jouy-en-Josas, France; 2 INRA, UR 631, 31326 Castanet-Tolosan, France

## Abstract

**Background:**

Ranks have been used as phenotypes in the genetic evaluation of horses for a long time through the use of earnings, normal score or raw ranks. A model, ("underlying model" of an unobservable underlying variable responsible for ranks) exists. Recently, a full Bayesian analysis using this model was developed. In addition, in reality, competitions are structured into categories according to the technical level of difficulty linked to the technical ability of horses (horses considered to be the "best" meet their peers). The aim of this article was to validate the underlying model through simulations and to propose a more appropriate model with a mixture distribution of horses in the case of a structured competition. The simulations involved 1000 horses with 10 to 50 performances per horse and 4 to 20 horses per event with unstructured and structured competitions.

**Results:**

The underlying model responsible for ranks performed well with unstructured competitions by drawing liabilities in the Gibbs sampler according to the following rule: the liability of each horse must be drawn in the interval formed by the liabilities of horses ranked before *and *after the particular horse. The estimated repeatability was the simulated one (0.25) and regression between estimated competing ability of horses and true ability was close to 1. Underestimations of repeatability (0.07 to 0.22) were obtained with other traditional criteria (normal score or raw ranks), but in the case of a structured competition, repeatability was underestimated (0.18 to 0.22). Our results show that the effect of an event, or category of event, is irrelevant in such a situation because ranks are independent of such an effect. The proposed mixture model pools horses according to their participation in different categories of competition during the period observed. This last model gave better results (repeatability 0.25), in particular, it provided an improved estimation of average values of competing ability of the horses in the different categories of events.

**Conclusions:**

The underlying model was validated. A correct drawing of liabilities for the Gibbs sampler was provided. For a structured competition, the mixture model with a group effect assigned to horses gave the best results.

## Background

Ranks in competitions have been used in genetic evaluation of sport and race horses for a long time. Langlois [1] used transformed ranks to predict breeding values for jumping horses. Ranks were used through earnings; these are, roughly, a transcription of ranks into a continuous scale. Later, Tavernier [2,3], inspired by the model proposed by Henery [4] for races, used a model including underlying liabilities (" underlying model" hereinafter). This model explains the ranks as the observable outcome of a hierarchy of underlying normal performances of horses in competition. These underlying performances serve to estimate breeding values for jumping horses. The parameters of this model were difficult to compute (numerical integration has to be used), and thus simpler models were proposed with different transformations of ranks, like the squared root of ranks [5], Snell score [6] or normal scores [7]. These became the most frequent criteria used in Europe for sport horse breeding value prediction [8]. These secondary approaches are similar to the direct use of discrete numerals instead of underlying liabilities in the analysis of discrete variables [9]. Still, the model with underlying liabilities seems to be the most appropriate. In its original formulation, variance components [2,3] were estimated by the joint mode of their marginal posterior distribution. This might be inappropriate with low numbers of data per level of effects, because numerical computations rely on some asymptotic approximations. Recently, Gianola and Simianier [10] proposed a full Bayesian approach to estimate variance parameters for the underlying model for ranks (the so-called Thurstonian model), where computations are achieved via MCMC Gibbs samplers.

In Gianola and Simianer [10], "events" were included as linear effects underlying the liability. However, it is easy to see that event effects, even if they are real (say, some tracks are more difficult than others) do not affect ranks, just because ranks are relative performances from one horse to another; this will be argued verbally and formally later. Thus, for rank analysis, event effects *do not exist*. However, it is well known that competitions are structured, and horses considered to be the "best" go to the "best" races and meet their peers who are supposed to be the "best". This causes a disturbance in predicting breeding values.

The aim of this paper was to validate the performance for genetic evaluation of the Bayesian approach in finite samples, and in particular the Gibbs sampler, through simulations. The criteria that we have considered are those usually found in horse breeding evaluation: fit to a normal score, raw ranks, and the proposed underlying model for ranks. Further, a second aim was to suggest a better model for structured competitions organised into different technical levels, as they really exist and is explained above.

## Analysis of ranks

### Model with underlying liabilities responsible for ranks

Data from sport competitions or races are the ranks of the horses in each event. The model used to analyse these results includes an underlying variable responsible for ranks. Let **y**_**k **_be the vector of ranking in the race *k *(or jumping event) and **y **the vector of complete data, i.e. all ranks in all events  with *m *the total number of events. Suppose an underlying latent variable *l *responsible for ranks, which follows a classical animal model:(1)

where *i *is the horse, ***β ***fixed effects, **a **vector of random additive genetic effects, **p **vector of random permanent environmental effects (common to the same horse for different events), **h **vector of random event effects, **e **vector of residuals and **x**_**ik**_, **z**_**ik**_, **w**_**ik **_incidence vectors. Let us note:

The conditional probability of a particular ranking in one race *k *is given by:(2)

where (*j*) is the subscript of horse ranked *j *in the race *k*, *n*_*k *_the number of horses present in the event *k *and *ϕ *the density of standard normal distribution. For complete data:

### Joint posterior distribution

Define Θ = [***β'***, **a'**, **p'**, **h'**] a vector of location parameters and , a vector of variance parameters. The residual variance  was fixed to 1 to achieve identifiability, since liabilities were on an unobservable scale. The density of the joint prior distribution of Θ and Λ has the form [10]

Above, *p *(|*ν*_*t*_, *S*_*t*_^2^) is the density of a scaled inverted chi-square distribution on *ν*_*t *_degrees of freedom, with *S*_*t*_^2 ^interpretable as a prior guess for  and *H *= [*σ*_*β*_, *ν*_*a*_, *ν*_*p*_, *ν*_*h*_, *S*_*a*_^2^, *S*_*p*_^2^, *S*_*h*_^2^] is a set of known hyper-parameters. **A **is the relationship matrix. The density of the joint posterior distribution is then(3)

### The Gibbs sampler

The Bayesian analysis and the Markov chain Monte Carlo sampling were performed according to Gianola and Simianer [10] except for the drawing of liabilities. The parameter vector was augmented with the unobserved liabilities, the location parameters Θ were drawn from multivariate normal distributions, and conditional posterior distributions of the dispersion parameters were scale inverted chi-square. Flat priors were used for fixed effects and variance components. The suggested procedure to draw liabilities in Gianola and Simianer [10] was the following:

1. drawing of the liability  of the last horse ranked from 

2. drawing of the liability  of the horse ranked just before the last one from a truncated normal distribution 

3. etc.

In fact, this algorithm is not a correct Gibbs sampler, and indeed did not converge in practice to correct rank statistics. The reason is that in step (1), for a Gibbs sampler, the liability  above has to be conditioned on all other parameters of the model, including information from the other horses. At step (1) this information exists from a previous MCMC cycle and is condensed in the liability of the previous horse,  so that .

The correct procedure is thus the following:

1. drawing the liability  of the last ranked horse in the interval] - ∞,  [, i.e. a lower liability than the liability of the horse ranked just before in the previous MCMC cycle, so in the truncated Normal distribution: 

2. drawing the liability  of the horse ranked just before the last one in the interval given by liabilities of the last horse ranked and two before the last:  so in the truncated Normal distribution: 

3. etc.

The marginal density of each liability knowing all other parameters was therefore the probability to be between the liability of the horse ranked before and the liability of the horse ranked after the particular horse and not only the probability to be before the particular horse. These drawings must be performed several runs to converge to the joint distribution, i.e. a set of liabilities which corresponds to the overall ranking of the event. The use of a previous drawing from the preceding iteration accelerates the convergence. This procedure was validated by checking the distribution of performances obtained: their mean and variance must correspond to the mean and variance of order normal statistics when the underlying model involved the same *μ*_*i *_for all horses. These moments are available in usual statistical libraries.

The core of the program was the TM software developed by Legarra [11] where drawing of liabilities according to ranks were added.

### The event effect

Competition in jumping as well as in races is structured according to the technical level of the event, for example the height of the obstacles and their positions. A natural choice to take into account the differences between events is to include an event effect as in model (1). The event is conceived as having a true additive effect on the underlying scale. Whereas this might be true, this is irrelevant as far as only ranks are analyzed. Consider for example a race with effect 0 where times to arrival were 20, 10 and 30 s. Rank is of course 2, 1, 3. Now assume that race had a true effect of 5, everything else being identical. Times were 25, 15, 35 and ranks were identical. Therefore, event has no effect on ranks, and there is no way of estimating an event effect from rank information. Thus, it might be fixed to zero to achieve identifiability with no loss of information. This will be demonstrated now. The probability of the ranks observed in an event given the parameters (eq. 2) can be rewritten as [12]:(4)

with *t*_*j *_= *l*_(*j*) _- *l*_(*j*+1)_, **V **the covatiance matrix with *v*_*i*, *i *_= 2, *v*_*i*, *i*+1 _= *v*_*i*, *i*-1 _= -1 and *v*_*i*, *j *_= 0 for all other *i*, *j*, and *v*_*j *_= *μ*_(*j*) _- *μ*_(*j*+1) _for *j *= 1, ..., *n*_*k *_-1. So that, for *j *= 1, ..., *n*_*k *_- 1:

Since the event effect is the same for all horses in the same event, it disappears from *ν*_*j*_:

As a result, the probability of the ranks observed in an event given the parameters is independent of the event effect so that the joint posterior distribution only depends on the prior distribution of the event effect. The event effect is, as a consequence, not identifiable, whatever the distribution of other effects (especially genetic effects) in the event. This is the same for all fixed or random effects which have the same effect on all horses in the event, for example a category of event effect. The presence of genetic effects (as sires) cross classified with events do not change this fact. So, an equivalent model to (1) is the following:(5)

### How to take into account differences between events: the mixture model

The reasoning that was followed in this work to include some effect linked to the competition effect is somewhat different from the event effect. Since competition is structured according to the technical level of the event, several categories of events are defined from the low level to the high level. Horses participate in the different categories roughly according to their expected competing abilities (genetic and environmental ones), with, of course, incertitude. Thus, the relationship between the true ability of the horse and category is not complete. The idea is to attribute a group to those horses that follow more or less the same circuit, i.e. roughly the same number of events in each category. The group is linked to the horse rather than to the event and so, in the same event, horses from different groups may meet. This makes it possible to estimate the effect, even if horses of different groups meet less often than horses of the same group, by definition. Thus, horses belong with some probability to different groups. This can be applied to genetic effects as well as permanent environmental effects. Therefore, the sum of the genetic and permanent environmental effects of a horse has the following *a priori *mixture distribution:(6)

where *n*_*g *_is the number of groups with *a priori *expected values *g*_*i *_and probabilities of assignment to a group *q*_*i*_. Performances thus follow a mixture of normal variables of these different groups with the same variance but different means. So, the group effect has a genetic interpretation and depends on the horse, not on the event. Therefore, it is the same for the horse across all its competing events, which is not the case for the simple "event" effect. A full analysis would compute posterior probabilities for *q*_*i*_, by MCMC or Expectation-Maximization algorithms. For simplicity, in this paper, a horse was assigned *a priori *to a group without computing the *q*_*i*_, according to the frequency of the different categories performed by the horse during the period studied. Therefore, because horses in the same event may have participated in competitions of different levels of competition and so belong to different groups, the group effect may be identified in (2) and (3). In the following, this model will be referred to as the mixture model.

### Simulations

The objective of this paper was to check if, by using the underlying model and computations as in [10], ranks are suitable phenotypes to estimate the aptitude of the horse to compete: genetic and environmental abilities.

For this work, and without loss of generality, the distinction between genetic and environmental effects is not necessary to verify the model, since all previous formulas have been derived with the complete model, showing no influence of distribution of genetic and environmental effects on the probability of ranking of an event. Further, the fact that horses have repeated performances provides the connections across events and categories and with other horses and, in that sense, the model with repeatability compares to a sire model with unrelated sires.

So, for simplicity, we simulated the so-called "competing ability" **c**, which can be seen as the sum of random additive genetic plus permanent environmental effects, *c*_*i *_= *a*_*i *_+ *p*_*i*_. A horse population was simulated. The competing ability of the horse *i*, *c*_*i *_was drawn from the normal distribution assuming:

without any relationship between horses. Several performances were simulated for each horse. Residuals for each performance were drawn from a normal distribution with fixed residual variance of 1 ( = 1). The repeatability of performances was thus defined as the following:

The ranking was obtained by the hierarchy of performances in each event.

Two structures of competition were analysed: one where the distribution of horses among events was random and another one where, as it is in reality, different levels (3), i.e. categories of competition, were simulated. In the first structure, horses were assigned to events at random. In the second structure, the higher the simulated ability of the horse, the higher the probability to participate in the highest level. This pretends to mimic what happens in reality, where horses with "better" expected ability compete together in "better" races. To simulate such a situation, an estimated value of the competing ability of the horse was simulated with a supposed accuracy of  from the simulated true competing ability. Then according to these values, the rules of probability of Table 1 were used to assign horses into events with 3 different categories.

**Table 1:** Simulation of structured competition: probability of competing in the three categories

	Estimated competing ability		
Category	1/3 Lowest	1/3 Medium	1/3 Highest
1	90%	8%	2%
2	8%	84%	8%
3	2%	8%	90%

The simulated population included 1000 horses. Different numbers of horses per event and numbers of events per horse were simulated. For the unstructured competition, 10 to 40 performances per horse with 4 to 20 horses per event were simulated, with an equal or variable number for all events. For the structured competition, 10 to 50 events per horse were simulated with an equal number of horses per event (10). Each scenario was repeated 20 times except for the scenario with structured competition and 10 events per horse which was repeated 50 times.

### Model and criteria used in simulations

The first model used to estimate repeatability and competing ability of horses in simulations was the underlying model proposed in (1) in its equivalent form (4). The model was then:

Estimates were obtained with the Gibbs sampler from the joint posterior distribution in (3). The Gibbs sampler consisted of 1,000 iterations (with 150 of burn-in) with sampling of location parameters (***β***, ***c***) and variance components (, ). Within each iteration, 100 (only in the first iteration) or 10 iterations were run to draw liabilities. Autocorrelation between iterations were insignificant for lags greater than 13. Thus, samples were taken every 15 iterations. Convergence of chain was checked by the Geweke diagnostic [13]. In addition, three other models were used to analyse the simulated data. First, the simulated performances were analysed as a continuous trait; this provides an upper bound of the quality of the estimates because it is the best inference that could ever be done. Second, we included, for comparison with the underlying model, traditional measurements attributed to ranks in literature and used in genetic evaluation: raw ranks and normal scores. Normal scores are expected values of ordered multiple identical normal distributions. For these three pseudo-traits, a mixed linear model was used:

with *y*_*ik *_the normal score of horse *i *according to its rank and number *n*_*k *_of horses in the event or raw ranks (1,2, ..., *n*_*k*_). In the structured competition, normal scores were used first in a single trait model whatever category of event, and second, with a multiple trait model, i.e., one trait for each category of event. The estimates of repeatability were obtained with REML using SAS^® ^proc mixed [14] for the analysis of true underlying performances, normal score and ranks and by Gibbs sampling using one chain with 50,000 iterations for the normal score with the multiple trait model.

The last model was the mixture model proposed in the previous section. For the underlying mixture model the horse group was defined by the rounded mean value of grades affected to ordered categories of its competing events. For example: if there were 3 categories of competition with grades (1, 2, 3), a horse performed 10 events, 3 of grade 1, 2 of grade 2 and 5 of grade 3. This horse was assigned to the second group of horses because the mean value of the grades was 2.2. The model, written in terms of competing abilities, now becomes:

with ***κ ***the new vector of "competing ability" of the horse, a normal distribution defined as the following:

where **g **the vector of mean values of the 3 groups of horses, **w**_**i **_a design vector which allocated the group to the horse. So that the mean of the redefined "competing abilities" is:

with *q*_*r *_the proportion of each *n*_*g *_groups in population. The variance is:

Variance  includes extra variation due to equating a mixture by a linear expectation. The repeatability was defined as:

All parameters were estimated with the same Gibbs sampler as the first underlying model and **g **was estimated as a fixed effect.

## Results

### Validation of drawing of performances

As proposed in the method section, the algorithm used to draw performances knowing ranks was validated by comparing results with first and second moment of normal order statistics. The results are given in Table 2. For comparison, moments of normal scores were computed using sub-routines of NAG [15].

**Table 2:** Mean and Variance of drawn liabilities and of normal order statistics

Ranking	Mean		Variance	
	Drawing	Order Stat.	Drawing	Order Stat.
1	1.527	1.539	0.352	0.344
2	0.990	1.001	0.220	0.215
3	0.640	0.656	0.172	0.175
4	0.359	0.376	0.151	0.158
5	0.110	0.123	0.148	0.151
6	-0.136	-0.123	0.154	0.151
7	-0.385	-0.376	0.153	0.158
8	-0.665	-0.656	0.171	0.175
9	-1.008	-1.001	0.202	0.215
10	-1.538	-1.539	0.344	0.344

10 "equal" competitors by event, 1000 repetitions, 100 iterations for each event

### Unstructured competition

Table 3 summarizes the results of simulations with different numbers of horses per event and different numbers of events per horse. The repeatability estimated was compared to the one obtained directly on the underlying performance as data. These results showed that the model and the procedures used to estimate parameters performed well: the estimates of repeatabilities were close to those simulated and regressions of competing ability of the horses on estimates were close to 1, as expected.

**Table 3:** Estimate of repeatability for unstructured competition

Simulations						
Number of horses	1000	1000	1000	1000	1000	1000
Number of events	2500	1000	500	400+400+200	10000	2500
Number of events per horse	10	10	10	10	40	10
Number of horses per event	4	10	20	5/10/20	4	4
Total number of ranks	10000	10000	10000	10000	40000	10000
Simulated repeatability	0.25	0.25	0.25	0.25	0.25	0.10
**Repeatability estimated**						
True underlying performance	0.251	0.249	0.251	0.249	0.251	0.100
Ranks and Underlying model	0.251	0.252	0.253	0.248	0.253	0.099
Normal Score	0.145	0.199	0.222	0.196	0.144	0.057
Raw ranks	0.144	0.197	0.218	0.068	0.144	0.057

**Standard deviation of repeatability over replicate**						
True underlying performance	0.009	0.012	0.012	0.011	0.007	0.007
Ranks and Underlying model	0.010	0.015	0.012	0.011	0.007	0.007
Normal Score	0.007	0.012	0.011	0.008	0.004	0.004
Raw ranks	0.007	0.012	0.011	0.005	0.004	0.004

**Regression coefficient between simulated and estimated competing ability**						
True underlying performance	0.997	1.006	1.004	0.992	1.003	1.014
Ranks and Underlying model	0.998	0.997	1.004	0.996	1.004	1.013
Normal Score	1.406	1.160	1.088	1.157	1.413	1.374
Raw ranks	1.408	1.169	1.101	2.696	1.414	1.374

20 replicates of each simulated scenario

The same simulations were used to estimate competing ability of the horses using the other traditional criteria in horse breeding evaluation. All traditional criteria, (Table 3) underestimated the repeatability, especially when a variable number of horses per event was simulated. According to the standard deviation between replicates, the differences between simulated and estimated repeatability were still significant with 20 horses per event. Thus, there is a great loss of information by using normal scores or raw ranks.

### Structured competition

The probability law used to construct the structured competition gave the proportions of horses in the different levels of competition reported in Table 4 (averages over 50 replicates). These proportions were similar to those obtained in jumping competition in France for example (if dividing the level of competition into 3 parts). Thus, these simulations mimicked real data well.

**Table 4:** Mean of the number of horses that participate almost once in different levels of competition

	Level category		
Level category	1	2	3
1	604.0	430.4	234.3
2	430.4	724.5	421.6
3	234.3	421.6	578.9

50 replicates, 10 events per horse

In this case (Table 5), with the underlying model for the ranks, repeatability was clearly underestimated (0.184 versus 0.250 simulated) due to underestimation of the differences between the average values of competing abilities of horses that participated in different categories of competitions (Table 6). This is because the assumption of normality of competing abilities tends to shrink these differences towards 0. This bias decreases with more information, but even with a very large number of events (50) per horse, the estimates of repeatability are still biased (0.215). The other criteria also underestimated the repeatability even more than the underlying model for ranks and, on the contrary, with no decrease of bias for increasing number of events per horse. With the multiple trait model, as in the single trait model, the repeatability was always underestimated, and the differences of average values of horses in each level were still underestimated. So, this model is not well suited to a structured competition.

**Table 5:** Estimates of repeatability for structured competition (3 categories)

	**10 events/horse^*a*^**		**50 events/horse^*b*^**	
		
	**Repeatability**	**Standard Deviation**	**Repeatability**	**Standard Deviation**
		
True underlying performance	0.249	0.012	0.248	0.008
Normal score single trait	0.134	0.008	0.134	0.007
Normal score multiple trait 1	0.151	0.019	0.171	0.009
Normal score multiple trait 2	0.145	0.018	0.171	0.010
Normal score multiple trait 3	0.158	0.017	0.177	0.011
Underlying model	0.184	0.011	0.217	0.009
Underlying mixture model	0.253	0.016	0.247	0.009

simulated repeatability 0.25

^*a*^50 replicates, ^*b*^20 replicates

**Table 6:** Estimates of competing ability according to category of events: means by category

	**10 events/horse^*a*^**			**50 events/horse^*b*^**		
		
	**Category 1 versus 2**	**Category 3 versus 2**	**s.d.**	**Category 1 versus 2**	**Category 3 versus 2**	**s.d.**
		
Number of ranks	3388/3314	3298/3314	129	16711/16823	16467/16823	640
Simulated values	-0.395	0.384	0.021	-0.380	0.389	0.024
Normal Score	-0.041	0.042	0.005	-0.064	0.067	0.004
Normal Score multiple trait 1	-0.070	0.060	0.009	-0.175	0.170	0.033
Normal Score multiple trait 2	-0.065	0.066	0.010	-0.175	0.173	0.034
Normal Score multiple trait 3	-0.056	0.072	0.010	-0.176	0.178	0.034
Underlying model	-0.100	0.102	0.012	-0.265	0.272	0.016
Underlying mixture model	-0.388	0.394	0.032	-0.377	0.382	0.022

^*a*^50 replicates, ^*b*^20 replicates

**Figure 1:**
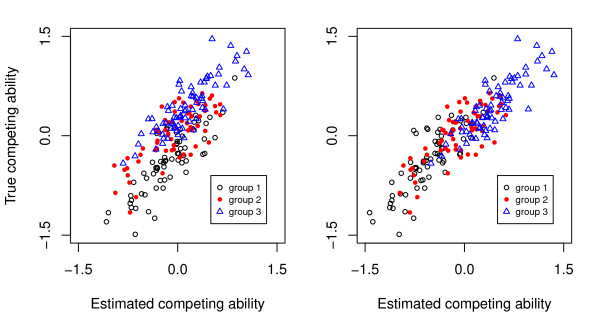
**True and estimated competing ability, underlying model for ranks (left), underlying mixture model for ranks (right)**.

Estimates with the mixture model are also shown in Tables 5, 6 and 7. Even with a low number of events per horse (10), repeatability was close to the value estimated from true underlying performances (0.253 versus 0.250). This better estimation was due to a better estimation of average values of competing ability of horses in each category of event (Table 6) and thus, in each defined group of horses (Table 7). This is shown in Figure 1, where solutions are plotted against true values (75 horses randomly selected from each group). The model with the underlying variable responsible for ranks gave a superposition of values in each group of horses whereas the mixture model gave a hierarchy between groups.

**Table 7:** Estimates of competing ability according to group of horses: means by groups

	10 events/horse^*a*^			50 events/horse^*b*^		
		
	Group 1 versus 2	Group 3 versus 2	s.d.	Group 1 versus 2	Group 3 versus 2	s.d.
Number of horses	330/337	334/337	15	329/338	334/338	15
Simulated values	-0.447	0.445	0.023	-0.431	0.449	0.026
Normal Score	-0.050	0.054	0.007	-0.074	0.078	0.006
Normal Score multiple trait 1	-0.083	0.073	0.011	-0.200	0.196	0.038
Normal Score multiple trait 2	-0.076	0.080	0.012	-0.200	0.200	0.039
Normal Score multiple trait 3	-0.063	0.090	0.012	-0.201	0.206	0.039
Underlying model	-0.116	0.123	0.015	-0.302	0.314	0.019
Underlying mixture model	-0.439	0.456	0.039	-0.428	0.441	0.026

^*a*^50 replicates, ^*b*^20 replicates

## Discussion

### Summary of results

The results validate the underlying model responsible for ranks used to measure performances in competition [2,3] as long as there is a correct estimation of parameters via the MCMC algorithm. The new algorithm proposed to draw underlying performances in agreement with ranking gave satisfactory results. Convergence may be accelerated by best sequences in the successive Gibbs sampler steps. However, our implementation was sufficient to give correct results for unstructured competition: correct repeatabilities and regression coefficients of 1 of true or estimated values for horses.

All other criteria for estimating breeding values and variance components underestimated the repeatabilities, in particular when the number of horses per event was variable, because in that case, the supposed variance in each event is largely conditioned by the trait chosen (normal score or ranks). All these results were validated by the repeatability obtained from the true underlying performance, which is the best possible inference that could ever be done.

With a structured competition, the underlying model with no mixture required a very large number of events per horse in order to have a large enough number of comparisons between horses of different levels to converge to the simulated repeatability, because these meetings are rare in structured competition, by definition. So, in practice, the mixture model developed is the best, also because it does not need a large number of events per horse.

### An explanation for the low heritability found in the literature for the ranking trait

Low heritabilities of traits related to ranking in jumping can be found in the literature: from 0.05 to 0.11 for those used in official breeding evaluation [8]. These values come from various studies. In Germany, for the squared root of rank, Luhrs-Behnke et al. [16] found 0.03. Higher estimates were obtained with the logarithm of earning in each event (with an event effect, so corresponding to a linear function of rank): 0.09 [17]. In Ireland and Belgium, normal scores were used as different criteria according to category of event and low heritabilities were also estimated: from 0.06 to 0.10 [18,19]. A higher heritability was found by Tavernier [20]: 0.16 with an underlying model, but employing a sire model and an estimation based on the mode of the marginal posterior distribution of the variances.

These results are in agreement with ours. Criteria related to ranks, used as raw data, underestimate the horse variance. The same will happen including a genetic effect and as a consequence the heritability of the underlying performance will be underestimated. This is similar to what happens in the threshold model, where the heritability in the observed scale is lower than that in the underlying scale and not invariant to transformation [21]. These results are an illustration of a scale problem and unsuitable models rather than a low heritability of jumping ability as often postulated [22] The most recent proposition to deal with structured competition was the use of normal scores with multiple traits according to categories but it did not perform well in our simulations. With the appropriate model, i.e. the underlying mixture model, higher heritabilities should be found in real data analysis.

### The mixture model

The sport competition or race programs are always structured in different categories according to the level of technical difficulty. So, there have to be differences between the means of the true underlying performances obtained in these different categories, whatever the ranking. These differences between means of performances can not be estimated by an event effect when ranks are the only phenotype available. We have shown that this is because such an effect is not involved in the probability function of the ranking in one event conditional on the parameters in the model. One could expect that the comparisons between horses in lots of events would enable to correctly estimate the genetic as well as the environmental effect and then, that the averages of genetic and environmental effects in each event are correct. But in fact, even with 50 events, the repeatability was underestimated.

Adding genetic effects through the use of the relationship matrix would have the same influence as increasing the number of events per horse: increasing the number of comparisons between horses. With a genetic effect, horses that do not compete in the same events may be compared through their relationship. However, the problem still exists: the best genetic values and the best sires will compete in the highest level of competition. So even if genetic links allow more comparisons, the problem of non-random allocation to categories of events remains. It will never be possible to ascertain that the number of comparisons will be sufficient to reach the correct values since this depends on the distribution of sires across categories of competition.

The aim of this study was not to estimate the level of connectedness necessary to estimate correctly genetic values but to correctly implement the model to analyze the phenotype (ranks) recorded and used to estimate breeding values. Adding groups of horses in the mixture model seems to give the suitable response. By adding an estimable effect, linked to the categories of event but not confounded with it, representing a summary of possible comparisons between categories of event, the phenotype is correctly modeled. Then, whatever the other effects are in the model, supposing different levels are present in at least some events, they will be correctly estimated, like the genetic effect.

In our simulations, the simplest method used to assign horses to categories was good enough to obtain good estimates of repeatability and moreover, good estimates of mean values of competing ability of horses in the different categories of events. A better model would fit a true mixture model by computing posterior estimates of assignment of animals to groups. In any way, this mixture model seems to be a good basis to improve the underlying model responsible for ranks to correctly account for the level of competition in the model.

## Conclusion

The full Bayesian analysis proposed by Gianola and Simianer of the Thurstonian model of Tavernier [2,3], i.e. the model of underlying unobservable liabilities responsible for ranks of an event, was validated. In addition, the algorithm in [10] for drawing conditional liabilities from ranks was corrected. In an unstructured competition, repeatability of performances was correctly estimated with this model. All other usual phenotypes such as normal score and raw ranks underestimated repeatability. For the realistic case of a structured competition, however, the underlying model model was unable to estimate the correct repeatability unless there was a cross-classified design of horses and categories of events. This does not happen in practice. Rather than trying to estimate an event effect, which makes no sense since these cannot be estimated, we suggest to use a mixture model assuming that *a priori *the horse population is a mixture. This model performed well, and the repeatability and the average level of each category of event were correctly estimated. More work must be done in the modelling of the mixture distribution.

## Competing interests

The authors declare that they have no competing interests.

## Authors' contributions

AR built the model and simulations and AL reviewed statistical concepts. AR implemented ranks specificities to the core of the Gibb sampler software provided by AL. AR and AL drafted the manuscript. All authors read and approved the final manuscript.
